# Microsatellite markers from the 'South American fruit fly' *Anastrepha fraterculus*: a valuable tool for population genetic analysis and SIT applications

**DOI:** 10.1186/1471-2156-15-S2-S13

**Published:** 2014-12-01

**Authors:** Silvia B Lanzavecchia, Marianela Juri, Angelica Bonomi, Ludvik Gomulski, Alejandra C Scannapieco, Diego F Segura, Anna Malacrida, Jorge L Cladera, Giuliano Gasperi

**Affiliations:** 1 Laboratorio de Genética de Insectos de Importancia Económica, Instituto de Genética 'Ewald A. Favret', CICVyA, Instituto Nacional de Tecnología Agropecuaria (INTA), Hurlingham, Buenos Aires, Argentina; 2 Consejo Nacional de Investigaciones Científicas y Técnicas, (CONICET), Ministerio de Ciencia, Tecnología e Innovación Productiva (MINCyT), Argentina; 3 Dipartimento di Biologia e Biotecnologie 'L. Spallanzani', Universitá di Pavia, Pavia, Italy

**Keywords:** Anastrepha fraterculus, microsatellite markers, genetic variability, SIT

## Abstract

**Background:**

*Anastrepha fraterculus *Wiedemann is a horticultural pest which causes significant economic losses in the fruit-producing areas of the American continent and limits the access of products to international markets. The use of environmentally friendly control strategies against this pest is constrained due to the limited knowledge of its population structure.

**Results:**

We developed microsatellite markers for *A. fraterculus *from four genomic libraries, which were enriched in CA, CAA, GA and CAT microsatellite motifs. Fifty microsatellite regions were evaluated and 14 loci were selected for population genetics studies. Genotypes of 122 individuals sampled from four *A. fraterculus *populations were analyzed. The level of polymorphism ranged from three to 13 alleles per locus and the mean expected heterozygosity ranged from 0.60 to 0.64. Comparison between allelic and genotypic frequencies showed significant differences among all pairs of populations.

**Conclusions:**

This novel set of microsatellite markers provides valuable information for the description of genetic variability and population structure of wild populations and laboratory strains of *A. fraterculus*. This information will be used to identify and characterize candidate strains suitable to implement effective pest control strategies and might represent a first step towards having a more comprehensive knowledge about the genetics of this pest.

## Background

The South American fruit fly *Anastrepha fraterculus *Wiedemann (Diptera: Tephritidae) is an important pest of commercial fruit in the American continent. In subtropical and temperate regions of South America, this pest shares its habitat with the Mediterranean fruit fly *Ceratitis capitata *Wiedemann (Diptera: Tephritidae), and both species cause significant economic losses in fruit-producing areas. The presence of these species limits access to international markets due to quarantine restrictions imposed by fruit-fly-free countries. In Argentina, the National Control and Eradication Program (PROCEM) acts to control pest fruit fly species by using the only currently available control measures for *A. fraterculus *which are traps and pesticides.

Biological studies on the reproductive behavior ([1,2] and references therein) and artificial rearing [3-5] of this species have yielded valuable information for the development of an environmentally safe control method such as the Sterile Insect Technique (SIT) [6,7]. The population structure of *A. fraterculus *is still poorly understood, so that the development of suitable molecular tools might greatly help in providing a more complete scenario for the effective implementation of control strategies against this pest.

*A. fraterculus *has been recently recognized as a complex of cryptic species [8-11]. In Argentina and southern Brazil, only one biological entity, described as *A. fraterculus *sp.1 by Goday et al. [11], is present [12,13]; this entity has been previously characterized using sequence data [14] and behavioral studies [15,16].

The population genetics of fruit flies has been described using inter alia, mitochondrial sequence polymorphisms [14,17-22] and intron sequence polymorphisms [23-25]. In the last decade, the development and characterization of simple sequence repeats (SSRs or microsatellites) from fruit fly species has increased rapidly [26-31]. Microsatellites have been extensively used to resolve related populations [32-35] and to describe population dynamics and colonization patterns [36-40]. These markers have also proven useful to study tephritid species where limited genetic information is available by fortuitous cross-species amplification [30, 41, 42 and references therein].

In *A. fraterculus*, the information about genetic aspects of its populations is limited. Alberti et al. (2002) [43] analyzed 9 to 11 Argentinean and one south Brazilian populations of *A. fraterculus *by using isozymes and PCR-RFLP from the large subunit ribosomal DNA (16S rDNA) in the mitochondrial genome. These authors observed no variation among the populations studied and concluded that the Argentinean and south Brazilian populations belong to a single biological species. Later, the same results were shown using mitochondrial Cytochrome Oxidase I (COI) polymorphisms analyzed by sequencing [14]. Ludeña et al. [44] studied phylogenetic relationships among Andean-Ecuadorian and other Neotropical populations of *A. fraterculus *and related species by sequencing two mitochondrial regions within the COI and Cytochrome Oxidase II genes. These authors found that Andean-Ecuadorian populations of *A. fraterculus *are homogeneous with respect to their mitochondrial genome and thus appear to be members of a single gene pool. Oroño et al. [45] used inter-simple sequence repeats (ISSRs) to study the genetic structure of sympatric populations of *A. fraterculus *from different hosts in northwestern Argentina and found strong host-mediated differentiation between populations.

Although dominant markers (as ISSRs) and mitochondrial DNA sequences have been useful to resolve patterns of population structure in *A. fraterculus*, the information provided is from delimited regions. Highly polymorphic markers such as microsatellites have proven useful in deep genetic studies of other Tephritidae species, as described above. The development of this kind of markers for *A. fraterculus *is needed to answer questions both at the intra-population level (e.g., assigning parentage and kinship relationship) and at the inter-population level (e.g., differentiation and population structure). Also, these molecular tools could be useful to elucidate species within the complex of cryptic species.

Here, we present the development of *A. fraterculus *sp.1 microsatellite markers and their first application to study the genetic diversity of wild and lab populations of this pest. The development of microsatellite markers for *A. fraterculus *represents a fundamental advance toward an integrated pest management of this species. This information may help develop environmentally friendly control strategies against this species, and may in turn help diminish the use of insecticides and toxic baits.

## Methods

### Insects

Laboratory insects were obtained from: 1) the *A. fraterculus *IGEAF strain kept at the National Institute of Agricultural Technology (INTA) (Hurlingham, Buenos Aires, Argentina); this colony was established in 2007 with approximately 10000 pupae and maintained for 56 generations under artificial rearing and, 2) the *A. fraterculus *IPCL strain kept at the Insect Pest Control Laboratory (FAO/IAEA Seibersdorf, Austria); this colony was established in 2005 with approximately 1000 pupae and maintained for at least 72 generations (MT Vera, personal communication). Both laboratory strains were not refreshed (i.e. no wild material was introduced to refresh the genetic background) and were derived from a semi-mass rearing colony kept at Estación Experimental Agroindustrial Obispo Colombres, Tucumán, Argentina, which was originally initiated in 1997 with wild pupae recovered from infested guavas (*Psidium guajava *L.) collected in the vicinity of Tafí Viejo, Tucumán, Argentina [5]. Strains were identified as *A. fraterculus *by Dr R. Zucchi and Dr V. Hernandez-Ortiz and no wild material has been introduced to refresh the strain [16].

Wild insects were collected from infested guava fruits sampled in Concordia (31°23′32″S 58°01′01″W) and Puerto Yeruá (31° 31′ 53.04″ S, 58° 0′ 55.08″ W) localities (Entre Ríos Province, Argentina). As these localities are 37 km apart, they are separated enough to be considered as different populations. Adult individuals were random sampled from the adult flies recovered from 10 guavas per tree (four guava trees from each locality) [46]. Immature stages were reared to adult stage under laboratory conditions. The insects were washed with TE buffer (10 mM Tris-HCl, 10 mM EDTA, pH 8) and stored at -20°C until DNA isolation.

### Construction and screening of microsatellite-enriched libraries

Genomic DNA from 10 adult individuals of the *A. fraterculus *IGEAF strain (5 females and 5 males) were isolated using the DNeasy Blood & Tissue kit (Qiagen, Valencia, CA, USA) and used to generate four genomic libraries enriched for CA, CAA, GA and CAT microsatellite motifs (Genetic Identification Services, Chatsworth, CA, USA) following the standard cloning protocol described by Murray et al. (2008) [47].

In order to select suitable polymorphic regions, 144 nucleotide sequences from the four libraries (75-83% enriched), containing di- or tri-nucleotide repeats, were analyzed. The percentage of enrichment was calculated based on the proportion of microsatellite sequences obtained from all the clones sequenced. To detect *a priori *polymorphisms (before PCR amplification) and to exclude sequences with nucleotide differences in primer recognition sites (more than 10 nucleotide changes, E Value >e-90), comparisons among nucleotide sequences in the database of 144 sequences were performed using BLASTN 2.2.22+ [48]. To exclude loci with similarities to transposable elements or other undesirable sequences, comparisons against all GenBank+EMBL+DDBJ+PDB sequences (but no EST, STS, GSS, environmental samples or phase 0, 1 or 2 HTGS sequences) were performed using BLASTN 2.2.24+ [49]. All the nucleotide sequences were submitted to GenBank (https://www.ncbi.nlm.nih.gov/genbank/index.html)[GenBank KJ619797 - KJ619940].

### Primer design and evaluation of *A. fraterculus *microsatellite markers

Primer sets were designed from the nucleotide sequences selected in the above-described step, using Primer 3 software (http://simgene.com/Primer3) (Additional file 1). Microsatellite regions were evaluated by PCR using total DNA of single flies as template. Total DNA was isolated from *A. fraterculus *adult individuals (IGEAF strain) based on the protocol described by Baruffi et al. [50].

Reaction mixtures in a final volume of 10 µl contained: 20 ng template DNA, 1-1.5 mM MgCl_2_, 1.5 mM dNTPs, 0.5 µM of each primer, and 0.5 UTaq DNA polymerase (Invitrogen, Carlsbad, CA, USA). The PCR cycling conditions were: 2 min at 95°C, followed by 30 cycles of 30 s at 95°C (denaturation), 30 s at the optimized annealing temperature (see Additional file 1), and 30 s at 72°C (extension). After cycling, the reactions were incubated at 72°C for 10 min. The cycling reactions were performed in a Mastercycler Gradient Eppendorf Thermo-cycler (Eppendorf, Hamburg, Germany) and in a MJ Research PTC 100 Thermocycler (MJ Research Incorporated, Watertown, MA, USA). The amplification products were separated by electrophoresis in 1.5% (wt/vol) agarose gel in 0.5X TBE buffer, stained with ethidium bromide [51], and 1-kb DNA ladder (Invitrogen) was used as a molecular weight marker. Primer sets with robust and specific amplification were further assayed for fragment length polymorphism across a minimum of 10 individuals (5 females and 5 males) from the *A. fraterculus *IGEAF strain.

Fragment length polymorphisms were detected in two ways. Several microsatellite loci were evaluated by automated capillary electrophoresis. PCR products obtained with 5´-labeled forward primers (TET, 6-FAM and HEX dyes; Sigma-Aldrich, UK) were run in an ABI 310 DNA Analyzer (Applied Biosystems, Life Technologies, MA, USA) with GeneScan 500 ROX Size Standard (Applied Biosystems). Alternatively, non-labeled markers were evaluated using electrophoresis in 6% polyacrylamide native gels stained with ethidium bromide (10 mg/ml); for details see "Dye" column in Additional file 1. For unlabeled markers, the polymorphism was defined as the presence of at least two alleles (bands of different size) when 10 samples from the *A. fraterculus *IGEAF strain were evaluated by electrophoresis in 6% polyacrylamide gels.

The most polymorphic markers were selected based on the good performance in the PCR assay and allele scoring and, after that, by focusing on the number of alleles detected.

### Polymorphism evaluation in *A. fraterculus *populations

A set of 14 microsatellite loci was selected for polymorphism evaluation in the two lab populations (IGEAF and IPCL) and the two wild populations (Concordia and Puerto Yeruá). DNA samples were obtained from about 30 *A. fraterculus *adult individuals (15 females and 15 males) from each population, using the protocol described above. The alleles were assessed using labeled forward primers and the standard PCR cycling described above. The labeled PCR fragments obtained were run in an automatic sequencer (ABI3130XL, Applied BioSystems). The results were processed using GeneMapper v3.7 or Peak Scanner v1.0 (Applied BioSystems) to assign the genotype to each sample at each locus. All allele scores were visually inspected. To eliminate or reduce the signal of confounding, nonspecific amplicons, some loci required reaction optimization.

## Data analysis

The genotypic data from the IGEAF strain (31 individuals), IPCL strain (30 individuals), Concordia (32 individuals) and Puerto Yeruá (29 individuals) were analyzed. Expected and observed heterozygosity and number of alleles at each locus were estimated using ARLEQUIN 3.11 [52]. Deviation from the Hardy Weinberg equilibrium (HWE) and linkage disequilibrium after Bonferroni corrections were tested using GENEPOP 3.4 [53]. The frequency of null alleles was estimated using Microchecker [54]. The inbreeding coefficient *F_IS _*and the degree of differentiation among populations analyzed as pairwise *F_ST _*values (Weir and Cockerham 1984) [55] and as genotypic differentiation by Fisher´s method (exact G test) were tested using GENEPOP 3.4. P values were estimated by the Markov chain algorithms.

## Results

We analyzed 144 *A. fraterculus *microsatellite regions obtained from four microsatellite-enriched libraries (36 nucleotide sequences from each library). After sequence analyses, 89 microsatellite sequences showed to be adequate in terms of good quality of sequences, the presence of repeated regions in the middle of the sequence and flanking regions suitable for primer designing. Fifty pairs of primers were designed and evaluated in at least 10 individuals from the *A. fraterculus *IGEAF strain (see details in Additional file 1). From the designed primers, some assays showed lack of PCR amplification. As we worked with good-quality DNA samples, we considered that the lack of amplification was due to the presence of nanosatellites (short repetitions of dinucleotides) in the primer sequence (not detected in the step of primer design), or to the location of the microsatellite in genomic regions of difficult access, or to the presence of secondary DNA structures that prevent PCR amplification. In other cases, it was not possible to perform allele scoring due to the lack of reproducibility in PCR amplification among samples, the presence of multiple peaks (or bands in polyacrylamide gels), and the complex pattern of peaks observed in capillary electrophoresis analysis (see details in Additional file 1).

In order to select the most polymorphic loci for population genetic studies, 21 microsatellite markers were tested in at least 20 individuals from the IGEAF strain. From the original set, the 14 most polymorphic loci were chosen and tested in about 30 individuals of the two laboratory strains (IGEAF and IPCL) and the two wild populations (Concordia and Puerto Yeruá) (Additional files 1 and 2).

Similar values of expected heterozygosity and number of alleles in laboratory strains and wild populations were found (Additional file 2), with relatively high genetic variation in wild populations (H_E _IGEAF = 0.62; H_E _IPCL = 0.60; H_E _Concordia =0.64; H_E _Puerto Yeruá = 0.64). No significant linkage disequilibrium was detected between genotypes at the 14 loci (*P *> 0.001, Fisher's exact test). The locus/population combinations that were not in HWE were not concentrated in any population or at any locus. The departure from HWE was mainly due to a deficit of heterozygote. The Microchecker 2.2.3 analysis showed a general excess of homozygotes and indicated the presence of null alleles that might explain the heterozygote deficiency observed. According with this result, populations are possibly in HWE for these loci (Additional file 2).

Analysis of genotypic frequencies across all loci for each pair-wise comparison (G Test, Fisher´s method) showed significant differences between all pairs of populations (*P*<0.05; see details of allelic and genotypic frequencies for each locus in each population in Table S1 and S2 in additional files 3 and 4, respectively). Pair-wise *F_ST _*values significantly differed from zero (Table 1). The highest level of genetic differentiation (*F_ST _*= 0.1309) was observed between Puerto Yeruá and IPLC, whereas the lowest value of differentiation (*F_ST_*= 0.0261) was observed between Concordia and Puerto Yeruá. *F_IS _*values showed low incidence of inbreeding in all populations (Concordia *F_IS _*= 0.13; IPCL *F_IS _*= 0.25; IGEAF *F_IS _*= 0.16; Puerto Yeruá *F_IS _*= 0.21).

**Table 1:** Pairwise *F_ST_* values (Weir and Cockerham 1984) for the four *A. fraterculus* populations analyzed.

Populations	IGEAF	IPCL	Concordia
IPCL	0.0368*		
Concordia	0.0426*	0.0870*	
Puerto Yeruá	0.0671*	0.1309*	0.0261*

*Statistical significance P < 0.05

## Discussion

In this study, we developed a set of microsatellite markers for *A. fraterculus*. These markers contribute to the characterization of specific regions (144 SSR sequences, GenBank AN KJ619797 - KJ619940) for the Brazilian-1 morphotype [12] or *A. fraterculus *sp. 1 [11]. The 14 selected microsatellite markers were useful to describe the genetic variability of two wild populations from Argentina and two laboratory strains. High values of expected heterozygosity and number of alleles in laboratory strains compared to wild populations have also been observed by Aketarawong et al. (2011) [56], who compared mass-reared *Bactrocera dorsalis *Hendel (Diptera: Tephritidae) and wild populations of this pest from SIT target and non-target areas of Thailand. In addition, the same authors observed that the mass-reared strain had a lower inbreeding coefficient (*F_IS_*) than the wild populations. These low values were justified by the periodic refreshment of the rearing strain with wild material. In contrast with this, the *A. fraterculus *lab strains studied here also showed low values of *F_IS _*but neither strain was refreshed with wild material. The maintenance of genetic variability observed in our lab strains must be considered for the development of mass-rearing strains for SIT and deserves further research to address the genetic mechanisms underlying the generation or conservation of genetic diversity in this species. As previously described by Hartl and Clark (1997) [57], genomic rearrangements, recombination, and mutations are considered main mechanisms for the generation or maintenance of the genetic variation. Recently, new genomic data provided evidence that balancing selection maintains genome-wide functionally important genetic variation within species and natural populations [58]. In addition, studies on inter-genomic epitasis have shown that inter-genomic interactions can promote the maintenance of polymorphisms that impact on fitness [59].

Differentiation between both laboratory strains (IGEAF and IPCL) was surprising, because they have the same origin (semi-mass rearing strain from Estación Experimental Ovispo Colombres, Tucumán, Argentina). In addition, we observed a higher inbreeding coefficient (*F_IS_*) in IPCL than in IGEAF. These results might be explained due to the differences in the time each strain has been reared under artificial conditions (the IPCL strain was established two years before the IGEAF strain) and also by the number of individuals that were used to establish the populations. Also, the genetic effect of selection and genetic drift could drive the allelic frequency toward the differentiation of these strains as is evidenced in general [57] and for insect species in particular [60-63].

The microsatellite markers developed here might represent a powerful tool for future studies about the analysis of ecological processes and behavioral traits that correlate with genetics in nature. We consider that this information could be useful in the investigation of genetic aspects of *A. fraterculus *populations maintained under experimental or mass-rearing conditions, as the analysis of the dynamics of change of the genetic variability across generations under artificial rearing. Similar analyses performed for other dipteran species [61-63] have revealed a loss of genetic diversity across generations as a consequence of the domestication process. The maintenance of genetic variability across generations in laboratory strains observed in the present study are in line with preliminary results obtained for a wild and lab populations of *A. fraterculus *during the first generations of the adaptation process. These results evidenced a loss of genetic diversity across generations in the wild population introduced to laboratory conditions and maintenance of variability in the adapted laboratory strain [64].

Microsatellite markers may also be helpful to elucidate the species status within the *A. fraterculus *complex of cryptic species [10,12]. In the present work, we developed microsatellites from the Argentinean population of *A. fraterculus *(as represented in the IGEAF strain), described as sp. 1 by Goday et al. [11] and Brazilian-1 morphotype by Hernández-Ortiz et al. [12]. Microsatellite markers proved to be useful to describe the genetic diversity within populations of this morphotype in Argentina and could be used to expand the analysis to other American populations. These markers may also prove to be useful to differentiate morphotypes of this complex, bringing insights into the genetic diversity, gene flow, colonization and dispersal patterns of this pest. In this sense, studies performed on other fruit fly species of economic importance [39,40,65] have shown the usefulness of microsatellites to assess population genetic aspects of these species. In addition, the markers developed here could also be useful for cross species amplification in the genus *Anastrepha *and other Tephritidae species where limited genetic information is available, as described for other fruit fly species [30, 41, 42 and references therein]. These molecular tools will be useful to be applied in a comprehensive investigation of the population diversity of an invasive pest of economic importance in the American continent and in the development and implementation of improved control strategies taking into account the genetic context of this species.

## Conclusions

A total of 144 *A. fraterculus *sp. 1 microsatellite sequences were generated and analyzed. Selecting 14 microsatellite loci was useful to analyze four populations of *A. fraterculus*. The study of the genetic variability both in lab strains and wild populations represents a first step to explore the genetic forces modulating the levels of genetic variability during artificial rearing in this species. The microsatellite markers developed here will provide valuable insights into the population genetics, colonization patterns and phylogenetic relationships of this species and into ecological strategies in the field. In addition, these tools characterize a source of polymorphic molecular markers for species identification in the *fraterculus *complex of cryptic species and could represent a valuable tool for the analysis of the genetic variability of other congeneric species.

The results provided here are of paramount importance for the integral genetic knowledge of *A. fraterculus*, and particularly to identify and characterize *A. fraterculus *candidate strains to be used in environmentally friendly control strategies against fruit fly pests, such as SIT, which allow diminishing the use of chemical control and toxic baits.

## Competing interests

The authors declare that they have no competing interests.

## Authors' contributions

SBL analyzed the SSR nucleotide sequences, characterized the microsatellite markers, performed most of the analyses described in the manuscript and drafted the largest part of the manuscript. MJ participated in the characterization of the microsatellite markers and carried out molecular genetic studies. AB participated in the characterization of SSR markers. LG participated in sequence analysis and helped to draft the manuscript. ACS participated in the drafting of the manuscript. DFS helped in the design of the study and to draft the manuscript. JLC participated in the design of the study and helped to draft the manuscript. AM and GG hosted a part of the research performed in their laboratory, participated in the design of the study and helped to draft the manuscript. All authors read and approved the final manuscript.

## Supplementary Material

Additional file 1 Basic characteristics and PCR amplification conditions of 50 *A. fraterculus *microsatellite loci. Detailed legend: N: number of individuals analyzed; Dye: fluorescent label incorporated in forward primers; T: optimized annealing temperature; [MgCl_2_]: optimized magnesium chloride concentration; Size: size of PCR product predicted by Primer 3 software. A: Number of alleles detected. NA: no PCR amplification observed; ND: number of alleles not determined; MP: multiple peaks or bands observed in agarose gels or capillary electrophoresis analysis; CP: complex peaks: complex pattern of peaks observed in capillary electrophoresis analysis, difficult allele scoring. * Primers designed by GIS Company using Designer PCR software v 1.03 (Research Genetics, Inc.). Click here for file

Additional file 2 Microsatellite analysis of wild populations and laboratory strains of *A. fraterculus*. Detailed legend: *N_a_*: number of alleles per locus; *H_O_*: observed heterozygosity; *H_E_*: expected heterozygosity; *Null*: estimated null allele frequency [66]; *value differs significantly from expectation under Hardy-Weinberg equilibrium (after Bonferroni correction). †Significant frequencies of null alleles (*P *< 0.05). A total of 122 individuals (31 individuals of IGEAF, 30 of IPCL, 32 of Concordia and 29 of Puerto Yeruá) were analyzed. Click here for file

Additional file 3 Table S1 Allelic frequencies obtained from GENEPOP for IGEAF and IPCL laboratory strains and Concordia and Puerto Yeruá wild populations (122 individuals) for each locus (14 loci). Click here for file

Additional file 4 Table S2 Observed and expected genotypic frequencies obtained from GENEPOP for each population (4 populations, 122 individuals) and locus (14 loci). Click here for file
